# Medical treatment of 55 patients with COVID-19 from seven cities in northeast China who fully recovered: A single-center, retrospective, observational study

**DOI:** 10.1097/MD.0000000000023923

**Published:** 2021-01-15

**Authors:** Lichao Fan, Huan Liu, Na Li, Chang Liu, Ye Gu, Yongyu Liu, Yu Chen

**Affiliations:** a Department of Tuberculosis; b Department of Respiratory; c Department of Central Laboratory; d Department of Thoracic Surgery, Shenyang Tenth People's Hospital, Shenyang Chest Hospital; e Department of Respiratory, Shenyang Sixth People's Hospital, Shenyang 110044, Liaoning, China.

**Keywords:** COVID-19, drug, medical treatment, SARS-CoV-2

## Abstract

Coronavirus disease 2019 (COVID-19) is an emerging disease caused by severe acute respiratory syndrome coronavirus 2; no specific effective medication to treat the disease has been identified to date. We aimed to investigate the administered medications and intervention times for patients who completely recovered from COVID-19.

This single-center, retrospective, observational study included 55 patients with COVID-19 who were transferred to Shenyang Sixth People's Hospital between January 20 and March 15, 2020. Data on demographics, symptoms, laboratory indicators, treatment processes, and clinical outcomes were collected. Administered drugs and intervention times were compared in 47 and 8 patients with mild and severe symptoms, respectively.

All 55 patients recovered. Fifty-three patients (96.36%) received antiviral therapy, including 45 in the mild group (median treatment: 14 days; 17 received umifenovir) and all 8 severe-group patients (median treatment: 17.5 days; 4 received lopinavir/ritonavir). Twenty-nine patients (52.72%) were administered antibiotics, including 21 in the mild group (median treatment: 13.5 days; 15 received moxifloxacin) and all 8 in the severe group (median treatment: 9 days; 2 received linezolid). Moreover, 7 patients (12.72%) were treated with glucocorticoids and 9 (16.36%) with immunomodulators.

Given the 100% recovery rate, early administration of antiviral drugs can be considered. Umifenovir may benefit patients with mild symptoms, while lopinavir/ritonavir may benefit those with severe symptoms. Prophylactic administration of common antibiotics may reduce the risk of co-infection. The use of glucocorticoids is usually not necessary. Randomized, double-blind, and controlled trials remain necessary for more accurate conclusions.

## 1 Introduction

Coronavirus disease 2019 (COVID-19) is highly contagious and spreads rapidly through human-to-human transmission.^[1]^ The pathogenesis of severe acute respiratory syndrome coronavirus 2 (SARS-CoV-2) infection remains unclear, and effective drugs and regimens for the treatment of COVID-19 have not been identified.^[2,3]^ In China, nationally recommended trial-based antiviral and other symptom-managing drugs are administered. As such, a retrospective review of the types and doses of clinical drugs, courses of treatment, and intervention times in patients cured of COVID-19 would be highly informative for treating patients with this disease worldwide.

Data on medications that were administered to patients who ultimately recovered from COVID-19 are scarce but crucial for clinicians. To that end, we aimed to investigate the administered medications and intervention times for 55 patients confirmed to have COVID-19 who completely recovered after being transferred to Shenyang Sixth People's Hospital, a designated treatment facility in Liaoning Province.

## 2 Methods

### 2.1 Study design and participants

We performed a single-center, retrospective, and observational study at Shenyang Sixth People's Hospital (Shenyang, Liaoning, China), a government-designated centralized medical facility for the treatment of patients with COVID-19 in Liaoning Province. All patients were from hospitals that received patients for initial COVID-19 treatment in 7 cities in Liaoning Province, including Shenyang Chest Hospital. According to the Interim Guidelines issued by the World Health Organization (WHO) on January 12, 2020,^[4]^ patient throat swabs and sputum samples were collected. Real-time reverse transcription polymerase chain reaction (RT-PCR) was used to detect the nucleic acid of SARS-CoV-2. We included all 55 consecutive patients with COVID-19 who were treated between January 20 and March 15, 2020; none were excluded. Patients were categorized into 2 groups: the “mild” group (with mild/moderate symptoms) and the “severe” group (those with severe/critical symptoms). These classifications were according to the criteria stated in the COVID-19 Diagnosis and Treatment Plan issued by the National Health Commission of the People's Republic of China.^[3]^

The study was reviewed and approved by the Ethics Committee of Shenyang Chest Hospital (approval number: KYXM-2020-001-01) and was also documented by the Ethics Committee of the Shenyang Sixth People's Hospital. The requirement for written informed consent was waived owing to the rapid development of the infectious COVID-19 disease.

### 2.2 Data collection

We reviewed clinical manifestations as well as laboratory and radiological findings of all enrolled patients and collected data that included age, sex, epidemiological history, past history, symptoms, complications, laboratory indicators, therapeutic drugs, and intervention time.

### 2.3 Outcomes

The endpoint was the total patient recovery rate; individuals who met the discharge criteria were included in the “recovered” statistics. These discharge criteria were consistent with the China's COVID-19 Diagnosis and Treatment Plan^[3]^ as follows:

1. body temperature returned to normal and remained so for at least 3 days;
2. respiratory symptoms were appreciably relieved;
3. pulmonary imaging showed a significant improvement in acute exudative lesions; and
4. nucleic acid tests of the sputum, nasopharyngeal swabs, and other respiratory specimens were negative twice consecutively following a minimum interval of 24 hours.

### 2.4 Statistical analysis

Given that the purpose of this study was to examine the clinical characteristics and drug administration data for patients with COVID-19, no formal hypothesis was established with which to calculate the optimal sample size. Continuous variables are expressed as means (standard deviations) or medians (interquartile ranges [IQRs]), while categorical variables are denoted as percentages.

## 3 Results

**Figure 1:**
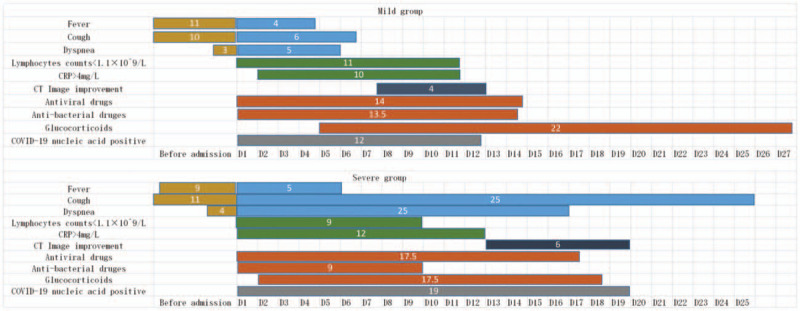
Clinical courses of major symptoms and medical treatment and duration of the viral shedding from illness onset in patients hospitalised with COVID 19. Figures show median duration of symptoms, abnormal laboratory indicators, and medical treatment. COVID-19 = coronavirus disease 2019, CRP = c-reactive protein, CT = computed tomography, D = days after illness onset.

The mean age of the 55 patients in our study was 46.8 years. Among them, 30 (54.55%) were male, 28 (50.91%) had been in Wuhan/Hubei, and 19 (34.55%) were complicated with other chronic diseases. Lung computed tomography scans showed local or diffuse infiltration shadows in 54 patients (98.18%), whereas the remaining patients (1.82%) had no inflammatory changes. There were 47 patients (85.45%) in the mild group and 8 (14.55%) in the severe group (Table 1). The most common symptoms of COVID-19 were fever (32 patients, 58.18%) and cough (27 patients, 49.09%). Seventeen patients (30.91%) were complicated with liver function impairment, 15 (27.27%) with hypoxemia, and 2 (3.64%) with acute respiratory distress syndrome (ARDS) (Table 2). The white blood cell counts, lymphocyte counts, and percentage of lymphocyte counts of patients in the mild group were in the normal range, although C-reactive protein levels (15.73 mg/L) were elevated. In the severe group, however, lymphocyte counts (0.78 × 10^9^ /L) and the percentage of lymphocytes (12.30%) were suppressed, while C-reactive protein levels (47.21 mg/L) were elevated (Table 2). In the mild and severe groups, the median durations for the lymphocyte counts to return to normal were 11 and 9 days, respectively; for those with lung shadows, marked improvements took on average 4 and 6 days, respectively. The time it took to achieve a negative COVID-19 RNA conversion was 12 and 19 days, respectively (Fig. 1).

**Table 1:** Epidemiological and baseline characteristics of 55 patients with COVID-19.

Clinical characteristics	Total (n = 55)	Mild group (n = 47)	Severe group (n = 8)
Age (yr)	46.36 ± 14.41	46·40 ± 14.70	46·12 ± 13.54
Sex (male/female)	30/25	24/23	6/2
Epidemiological history			
Have been to Wuhan/Hubei	28 (50.91%)	22 (46.81%)	6 (75.00%)
Have been in contact with confirmed cases	18 (32.72%)	16 (34.04%)	2 (25.00%)
Cluster onset	7 (12.72%)	7 (14.89%)	0
Other	2 (3.63%)	2 (4.26%)	0
Preexisting medical conditions			
Diabetes	8 (14.55%)	7 (14.89%)	1 (12.50%)
Coronary artery disease	3 (5.45%)	2 (4.26%)	1 (12.50)
Hypertension	8 (14.55%)	8 (17.02%)	0
Lung computed tomography scan			
Infiltration change	54 (98.18%)	46 (97.87%)	8 (100.00%)

COVID-19 = coronavirus disease 2019.

**Table 2:** Symptoms, complications, and laboratory test results of 55 patients with COVID-19.

Clinical characteristics	Total (n = 55)	Mild group (n = 47)	Severe group (n = 8)
Symptoms and signs			
Fever	32 (58.18%)	24 (51.06%)	8 (100%)
Headache	2 (3.64%)	2 (4.26%)	0
Cough	27 (49.09%)	24 (51.06%)	3 (37.50%)
Sore throat	7 (12.73%)	5 (10.64%)	2 (25.00%)
Fatigue	8 (14.55%)	8 (17.02%)	0
Shortness of breath	8 (14.55%)	5 (10.64%)	3 (37.50%)
Nausea and vomiting	4 (7.27%)	2 (4.26%)	2 (25.00%)
Diarrhea	6 (10.91%)	4 (8.51%)	2 (25.00%)
Muscle or joint pain	7 (12.73%)	5 (10.64%)	2 (25.00%)
Complications			
Acute respiratory distress syndrome	2 (3.64%)	0	2 (25.00%)
Hypoxemia	15 (27.27%)	7 (14.89%)	8 (100.00%)
Liver dysfunction	17 (30.91%)	11 (23.40%)	6 (75.00%)
Laboratory tests			
White blood cell count (×10^9^/L)	6.13	5.78	8.18
Lymphocyte count (×10^9^/L)	1.61	1.76	0.78
Lymphocyte percentage (%)	28.76	31.56	12.30
C-reactive protein (mg/L)	20.31	15.73	47.21
D-dimer (mg/L)	0.62	0.48	1.44
Procalcitonin (ng/mL)	0.08	0.07	0.11
Total bilirubin (μmol/L)	19.47	18.98	22.38
Alanine aminotransferase (U/L)	40.65	37.85	57.13
Creatinine (μmol/L)	54.25	54.13	55.00

COVID-19 = coronavirus disease 2019.

**Figure 2:**
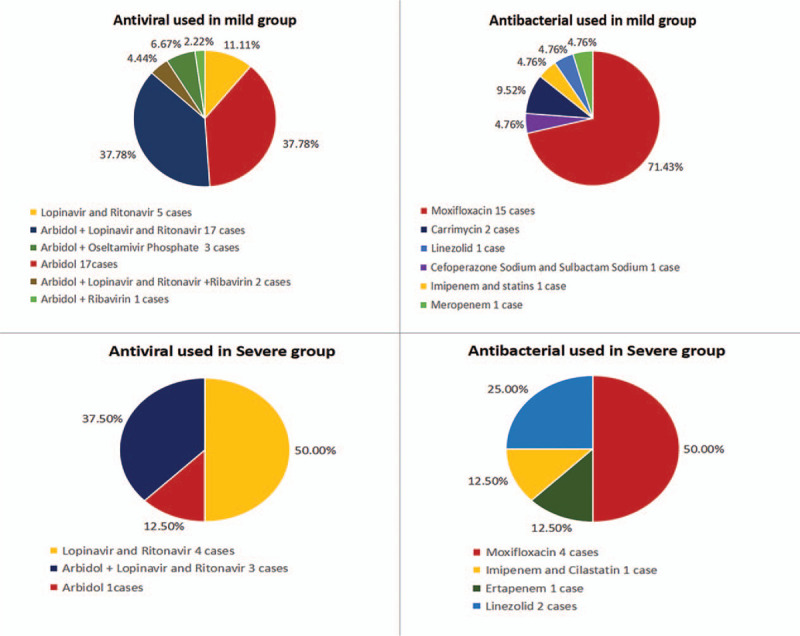
Use of antiviral drugs and antibiotics in mild and severe patients.

**Figure 3:**
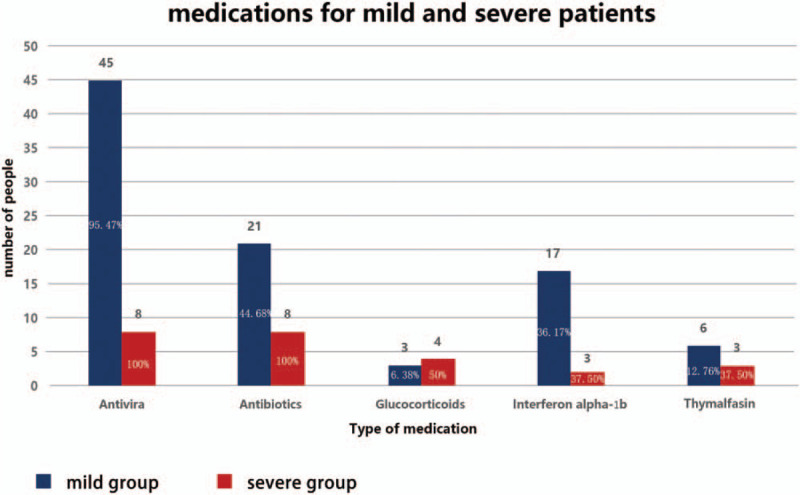
Medications for mild and severe patients.

Fifty-three patients (96.36%) received antiviral therapy for a median time of 14 days (IQR 12–18 days), while 2 patients (3.64%) were not administered antiviral drugs (1 was a pregnant woman and the other had asymptomatic infection). Among those who received antiviral drugs, 45 were in the mild group (95.74% of this group); their median treatment time was 14 days (IQR 12–17 days) and 17 of them (37.78%) were treated with umifenovir, 17 (37.78%) with umifenovir + lopinavir/ritonavir, and 5 (11.11%) with lopinavir/ritonavir. Moreover, all 8 patients in the severe group received antiviral drugs, with a median treatment time of 17.5 days (IQR 11–19.25 days). Four patients in the severe groups (50%) were treated with lopinavir/ritonavir, 3 (37.50%) with umifenovir + lopinavir/ritonavir, and 1 (12.50%) with umifenovir. Twenty-nine patients (52.72%) were treated with antibiotics for a median time of 10 days (IQR 8.5–15); 19 of these 29 patients (65.52%) were treated with moxifloxacin while 3 (10.34%) received linezolid. Among the patients treated with antibiotics, 21 were in the mild group (44.68% of this group); their median treatment time was 13.5 days (IQR 5.75–9.25 days) and 15 (71.42%) were treated with moxifloxacin while 2 (9.52%) received carrimycin. The remaining antibiotic recipients included all the 8 patients in the severe group (100%), with a median treatment time of 9 days (IQR 9.75–15.25); 4 patients (50%) were treated with moxifloxacin and 2 (25%) with linezolid. Seven patients (12.72%) were treated with glucocorticoids. Among the 7, 3 (6.38%) were in the mild group and 4 (50%) were in the severe group. Twenty of 55 patients (36.36%) received recombinant human interferon alpha-1b, including 17 patients (36.17%) in the mild group and 3 patients (37.50%) in the severe group; 9 (16.36%) were treated with thymalfasin, including 6 (12.76%) in the mild group and 3 (37.50%) in the severe group (Figs. 1–3).

## 4 Discussion

Patients with COVID-19 in the present study achieved a 100% recovery rate. Thus far, effective antiviral drugs to treat COVID-19 have not been identified, and opinions on whether antiviral drugs should even be used to treat COVID-19 differ.^[2]^ The National Health Commission of the People's Republic of China has repeatedly issued and revised the COVID-19 Diagnosis and Treatment Plan, which recommends antiviral drugs such as lopinavir/ritonavir, ribavirin, umifenovir, and alpha-interferon.^[3]^ In the present study, 53 patients (96.36%) received antiviral therapy early in the course of their disease for a median time of 14 days (IQR 12–18 days). In the mild group, 17 (37.78%) of the patients were treated with umifenovir (0.2 g orally once every 8 hours) and another 17 (37.78%) received a combination of umifenovir (0.2 g orally once every 8 hours) + lopinavir/ritonavir (400 mg/100 mg orally once every 12 hours) for a median time of 14 days (IQR 12–17 days). Liu et al^[5]^ described the effectiveness of umifenovir in the treatment of emerging respiratory infectious diseases such as influenza A (H1N1). Ji et al^[6]^ also confirmed the efficacy of umifenovir for the treatment of coronavirus infections in an in vitro study. Our present findings indicated that umifenovir might benefit patients with mild symptoms. In the severe group, 8 patients (100%) were administered antiviral drugs for a median time of 17.50 days (IQR 11–19.25 days); 4 (50%) were treated with lopinavir/ritonavir and 3 (37.50%) received a combination of lopinavir/ritonavir regimen. Lopinavir alone has poor bioavailability. Ritonavir can increase the plasma concentration of lopinavir by inhibiting CYP3A-mediated degradation of lopinavir in the liver.^[7]^ Nukoolkarn et al^[8]^ observed through molecular dynamics simulation studies that lopinavir/ritonavir can combine with the main protease 3CLpro of the severe acute respiratory syndrome (SARS) virus to achieve anti-coronavirus effects, but their affinity is not obvious. Chu et al^[9]^ found that lopinavir/ritonavir could inhibit coronavirus replication to some extent, thereby reducing the risk of ARDS or death in patients with SARS. Chan et al^[10]^ confirmed the efficacy of the combination of lopinavir/ritonavir and interferon-β for the treatment of Middle East Respiratory Syndrome Coronavirus (MERS-CoV) infection in animal models. In the present study, the use of lopinavir/ritonavir in the severe group was apparently effective in mitigating fever symptoms, promoting lung shadow absorption, and rapidly restoring the number of lymphocytes. The efficacy and safety of lopinavir/ritonavir are expected to be verified in future clinical randomized controlled trials. Ribavirin and interferon are also mentioned in the COVID-19 Diagnosis and Treatment Plan^[3]^; in the present study, 4 patients in the mild group (7.27%) were administered ribavirin combined with antiviral therapy. Nucleoside analogs theoretically ought to possess anti-coronavirus activity to a certain extent^[11]^; however, ribavirin was found to have a minimal antiviral effect against coronavirus in vitro.^[12]^ In the present study, 20 patients received aerosol inhalation of alpha-interferon soon after diagnosis (50 μg, twice per day). A retrospective study of patients with Middle East Respiratory Syndrome (MERS) in 2019 showed that interferon did not accelerate virus clearance,^[13]^ while a study of patients with SARS showed that alpha-interferon did not improve the patients’ prognosis.^[14]^ In the present study, a small number of patients were treated with ribavirin, and alpha-interferon was simply used to assist aerosol inhalation. Therefore, the usefulness of these 2 drugs for patients with COVID-19 is difficult to evaluate.

In terms of antimicrobial use, the WHO recommends empirical antimicrobial therapy based on the clinical diagnosis.^[4]^ China's COVID-19 Diagnosis and Treatment Plan^[3]^ also emphasizes the avoidance of blind or inappropriate use of antibiotics. Kim et al^[15]^ found that 38% of their patients with H1N1 infection developed secondary bacterial pneumonia 48 hours after admission to the intensive care unit, and that early empirical treatment helped improve their prognosis. Experience with SARS^[16]^ and MERS^[17]^ also suggests that prophylactic antibiotics may be appropriate after assessing the risk of co-infection in patients with severe symptoms.

Bacterial infection rates after SARS-CoV-2 infection remain unclear. In the present study, among 21 patients received antibiotics in the mild group, 12 (57.14%) patients were administered prophylactic drugs and 9 (42.86%) underwent empirical treatment (mainly with single-antibiotic moxifloxacin 400 mg ivgtt, qd); the possibility of atypical pathogenic bacteria (such as *Mycoplasma pneumoniae*) related infections was also considered. Epidemiological survey results in China show that *M pneumoniae* and *Streptococcus pneumoniae* are the main pathogens of adult community-acquired pneumonia in China. Because of their high resistance to macrolides, our guidelines recommend respiratory quinolone antibiotics usage.^[18]^ In the severe group, 8 patients received antibiotics, 5 (62.50%) patients were administered prophylactic medication, and 3 (37.50%) received empirical treatment. Prophylactic medication was administered to comorbid patients with diabetes, chronic lung diseases, and ARDS who were at the early stage of receiving glucocorticoids. In the severe group, patients with a relatively high risk of infection by drug-resistant bacteria (1 case each of prehospital antibacterial treatment ineffectiveness with ARDS, ventilator-associated pneumonia, and chronic structural lung disease with ARDS) were treated with broad-spectrum antibiotics. In consideration of influenza virus infection, *Staphylococcus aureus* and *S pneumoniae* are the main infections.^[19,20]^ The treatment plan is based on linezolid combined with cefoperazone and sulbactam or imipenem and cilastatin. One patient with ventilator-associated pneumonia had multi-drug-resistant *Klebsiella pneumoniae* in sputum culture and was administered meropenem. Gradually treatment was downgraded after symptoms improved. No evidence of secondary bacterial infection was observed in patients who received prophylactic medication. The present study suggested that early and prudent use of prophylactic antibiotics in patients with COVID-19 may help reduce the risk of co-bacterial infections. Empirical anti-infective treatment for severely ill patients under the pressure of high risk of drug resistance may benefit the prognosis of the disease.

The WHO does not recommend the systematic use of glucocorticoids for viral pneumonia or concurrent ARDS.^[4]^ China's COVID-19 Diagnosis and Treatment Plan recommends hormones as adjuvant therapy.^[3]^ In the present study, 48 (87.28%) patients did not receive glucocorticoids, while 7 patients (12.72%) received such agents during the rapid progression of their disease (i.e., respiratory failure and large area exudation in both lungs). Glucocorticoids were administered to inhibit inflammation and improve oxygenation at a dose of Methylprednisolone 1 to 2 mg/kg/day. Treatment was gradually reduced over 5 to 7 days until discontinuation; and they showed no adverse reactions. As such, glucocorticoids appear to be unnecessary for patients with mild manifestations of COVID-19, while their use in treating patients with severe disease is controversial.

Thymosin α1 is a thymosin hormone responsible for restoring the homeostasis of the immune system. It plays a key role in the development of thymocytes, as well as increases the resistance of thymocytes to glucocorticoid-induced death. There is evidence that thymosin α1 is used as an immune enhancer for SARS patients and can effectively control the spread of infection. According to the COVID19 treatment guidelines of the National Health Commission of China, the use of thymosin α1 may be an alternative treatment option for COVID-19 patients with low lymphocyte count or immunodeficiency.^[21]^ There are currently research reports that Thymosin α1 (Tα1) can restore reduced lymphocytes and improve the function of failed T cells, thereby reducing the mortality of severe COVID-19,^[22]^ In the present study, 9 (16.36%) patients were administered thymalfasin. Thymus Faxin is a chemically synthesized drug, similar to the human body's natural thymosin α1 in chemical structure and spatial structure with potential clinical application. This notion requires further clinical observation and study. While high-flow oxygen therapy, invasive mechanical ventilation, and extracorporeal membrane oxygenation were provided to patients in the present study, their use was not investigated.

Our findings suggest that, while specific antiviral drugs are yet to be developed, currently available antiviral agents should be considered when treating patients with COVID-19. The prophylactic administration of single antiviral drugs to patients with severe symptoms, as well as to a proportion of those with mild manifestations, may help reduce the risk of co-infection. However, the use of glucocorticoids and immunomodulators needs further study.

Our study had some limitations given its single-center, retrospective, and observational nature. Owing to its small sample size, only descriptive data were available, and no statistical analyses were performed. Hence, randomized, double-blind, and controlled trials remain necessary for more accurate conclusions. Nevertheless, our data ought to provide helpful preliminary information at this stage of the COVID-19 pandemic.

## Acknowledgments

We thank all patients involved in the study. We would like to thank Editage (www.editage.cn) for English language editing.

## Author contributions

Yu Chen and Yongyu Liu designed the study. Chang Liu and Na Li were responsible for the literature search. Lichao Fan and Ye Gu collected the epidemiological and clinical data. Huan Liu and Lichao Fan processed statistical data, Huan Liu and Yu Chen drafted the manuscript. Yongyu Liu revised the final manuscript.

**Conceptualization:** Yu Chen, Huan Liu, Yongyu Liu.

**Data curation:** Na Li, Chang Liu.

**Formal analysis:** Lichao Fan.

**Funding acquisition:** Yu Chen.

**Investigation:** Huan Liu.

**Methodology:** Lichao Fan, Chang Liu.

**Project administration:** Ye Gu, Yongyu Liu.

**Resources:** Ye Gu.

**Software:** Lichao Fan, Na Li.

**Supervision:** Na Li, Yongyu Liu.

**Validation:** Huan Liu.

**Writing – original draft:** Yu Chen, Huan Liu, Yongyu Liu.

**Writing – review & editing:** Yu Chen, Chang Liu, Yongyu Liu.

## Abbreviations

Abbreviations: ARDS = acute respiratory distress syndrome, COVID-19 = Coronavirus disease 2019, H1N1 = influenza A, IQRs = interquartile ranges, MERS = Middle East Respiratory Syndrome, MERS-CoV = Middle East Respiratory Syndrome Coronavirus, RT-PCR = reverse transcription polymerase chain reaction, SARS = severe acute respiratory syndrome, SARS-CoV-2 = severe acute respiratory syndrome coronavirus 2, WHO = World Health Organization.
